# Multi-omics analyses reveal *MdMYB10* hypermethylation being responsible for a bud sport of apple fruit color

**DOI:** 10.1093/hr/uhac179

**Published:** 2022-08-29

**Authors:** Yu Liu, Xiu-hua Gao, Lu Tong, Mei-zi Liu, Xiao-kang Zhou, Muhammad Mobeen Tahir, Li-bo Xing, Juan-juan Ma, Na An, Cai-ping Zhao, Jia-Long Yao, Dong Zhang

**Affiliations:** College of Horticulture, Yangling Sub-Center of National Center for Apple Improvement, Northwest A&F University, Yangling, Shaanxi, China; College of Horticulture, Yangling Sub-Center of National Center for Apple Improvement, Northwest A&F University, Yangling, Shaanxi, China; College of Horticulture, Yangling Sub-Center of National Center for Apple Improvement, Northwest A&F University, Yangling, Shaanxi, China; College of Horticulture, Yangling Sub-Center of National Center for Apple Improvement, Northwest A&F University, Yangling, Shaanxi, China; Tianshui Institute of Pomology, Tianshui, Gansu, China; College of Horticulture, Yangling Sub-Center of National Center for Apple Improvement, Northwest A&F University, Yangling, Shaanxi, China; College of Horticulture, Yangling Sub-Center of National Center for Apple Improvement, Northwest A&F University, Yangling, Shaanxi, China; College of Horticulture, Yangling Sub-Center of National Center for Apple Improvement, Northwest A&F University, Yangling, Shaanxi, China; College of Horticulture, Yangling Sub-Center of National Center for Apple Improvement, Northwest A&F University, Yangling, Shaanxi, China; College of Horticulture, Yangling Sub-Center of National Center for Apple Improvement, Northwest A&F University, Yangling, Shaanxi, China; The New Zealand Institute for Plant and Food Research Ltd, Private Bag 92169, Auckland 1142, New Zealand; College of Horticulture, Yangling Sub-Center of National Center for Apple Improvement, Northwest A&F University, Yangling, Shaanxi, China

## Abstract

Apple bud sports offer a rich resource for clonal selection of numerous elite cultivars. The accumulation of somatic mutations as plants develop may potentially impact the emergence of bud sports. Previous studies focused on somatic mutation in the essential genes associated with bud sports. However, the rate and function of genome-wide somatic mutations that accumulate when a bud sport arises remain unclear. In this study, we identified a branch from a 10-year-old tree of the apple cultivar ‘Oregon Spur II’ as a bud sport. The mutant branch showed reduced red coloration on fruit skin. Using this plant material, we assembled a high-quality haplotype reference genome consisting of 649.61 Mb sequences with a contig N50 value of 2.04 Mb. We then estimated the somatic mutation rate of the apple tree to be 4.56 × 10 ^−8^ per base per year, and further identified 253 somatic single-nucleotide polymorphisms (SNPs), including five non-synonymous SNPs, between the original type and mutant samples. Transcriptome analyses showed that 69 differentially expressed genes between the original type and mutant fruit skin were highly correlated with anthocyanin content. DNA methylation in the promoter of five anthocyanin-associated genes was increased in the mutant compared with the original type as determined using DNA methylation profiling. Among the genetic and epigenetic factors that directly and indirectly influence anthocyanin content in the mutant apple fruit skin, the hypermethylated promoter of *MdMYB10* is important. This study indicated that numerous somatic mutations accumulated at the emergence of a bud sport from a genome-wide perspective, some of which contribute to the low coloration of the bud sport.

## Introduction

Bud sports have been widely used for selection of new cultivars in fruit trees, including grape, peach, apple, and citrus [1]. Of various economic traits selected from bud sports, fruit skin color is a top trait [2], because it is more easily identified than other variations. As a result, numerous apple cultivars with superior color traits, such as more redness, have been developed. For example, the well-known apple cultivar ‘Red Delicious’ with bright red fruit skin was selected from ‘Hawkeye’ with red-and-gold striped skin [3]. From ‘Red Delicious’, four continuous generations of sport mutant cultivars have been selected with superior ability to develop red color on fruit skin, such as ‘Starking’, ‘Starking Double’, ‘Red King’, and ‘Oregon Spur’ [4]. ‘Oregon Spur II’ is the fifth-generation bud sport of ‘Red Delicious’, and is widely planted due to its superior color. However, reversion to green-skinned fruit on ‘Oregon Spur II’ trees has been discovered in China. Orchard households in China mostly pick scions without confirmation of the characteristics of the parent tree. The practice of grafting degenerated mutants causes significant economic losses. A better understanding of somatic mutations causing cultivar degeneration is important to maintain the traits of elite cultivars.

Accurate genome assembly is the basis of genome-wide gene function studies. Due to the high heterozygosity and repetitive sequences, assembling an accurate apple reference genome is a time-consuming and labor-intensive challenge [5]. Currently, high-quality reference genome assemblies of cultivated apples are developed using homozygous diploid or triploid plants [6, 7]. Furthermore, a phased haplotype reference genome was developed using a heterozygous diploid apple cultivar, ‘Gala’ [8]. As ‘Red Delicious’ is not directly related to ‘Gala’, a high-quality genome assembly of ‘Red Delicious’ is required for the study of the sport mutants of ‘Red Delicious’.

According to Muller’s ratchet theory, somatic mutations accumulate in non-recombining plants without sexual recombination [9]. Somatic mutations have been reported to accumulate during development in individual horticultural plants, such as peach, strawberry, and sweet orange [10, 11]. Previous studies have reported that the emergence of bud sports is caused by somatic mutations such as DNA sequence and epigenetic variation [2]. The insertion of a ‘redTE’ transposable element (TE) in the promoter of *MdMYB10* is associated with the red-skinned phenotype of apple [6]. In grape, the insertion of a Gret1 retrotransposon in the promoter of *VvmybA1* causes inactivation of the gene and thus the white color of the grape [12]. Furthermore, DNA methylation levels of the *MdMYB10* promoter are associated with the variable color patterns in apple [6, 13–15]. In pear, green-skinned mutants show hyper-methylation in the *PcMYB10* promoter compared with red pears [16, 17].

Fruit skin color is a key indicator of fruit quality with regard to commercial and nutritional value [18]. The redness of fruit skin is determined by the types and contents of anthocyanin. The mechanism of anthocyanin biosynthesis has been elucidated in *Arabidopsis thaliana*, as well as in fruit crops, like apple and pear [19–23]. Genes encoding key enzymes and transcription factors for anthocyanin biosynthesis have been identified in *A. thaliana* [24]. The expression of anthocyanin biosynthesis genes is determined by the MBW complex, consisting of three types of transcription factors, including MYB, bHLH, and WD40. The MYB proteins can directly bind to the promoter of the anthocyanin biosynthesis genes encoding chalcone synthase (CHS), chalcone isomerase (CHI), flavanone-3-hydroxylase (F3H), flavonoid 3′-hydroxylase (F3′H), and flavonol synthase (FLS) [25, 26]. The key MYB regulator in apple anthocyanin biosynthesis is MdMYB10 [27], which has two other alleles, named MYB1 [20] and MYBA [28, 29]. MYB1/10/A promote anthocyanin accumulation by activating anthocyanin synthesis genes encoding dihydroflavonol 4-reductase (DFR), anthocyanidin synthase (ANS), UDP-glucose:flavonoid 3-glucosyltransferase (UFGT), and glutathione *S*-transferase (GST) [15, 20, 27, 28, 30].

This study focused on the somatic mutations accumulated at the emergence of a bud sport in a 10-year-old tree of ‘Oregon Spur II’. To identify the genetic and epigenetic mutations underpinning this bud sport, we assembled a reference genome of ‘Oregon Spur II’ and used this reference genome to carry out integrative genomic and transcriptomic analysis to compare the mutant branch with the original type branch. The present study aimed to answer the following questions. What are the characteristics of somatic mutations and gene expression mutations when bud sports appear in an apple tree? What is the main factor resulting in the low-coloration bud sport?

## Results

### Characterization of an ‘Oregon Spur II’ bud sport with reduced red coloration on fruit skin

**Figure 1:**
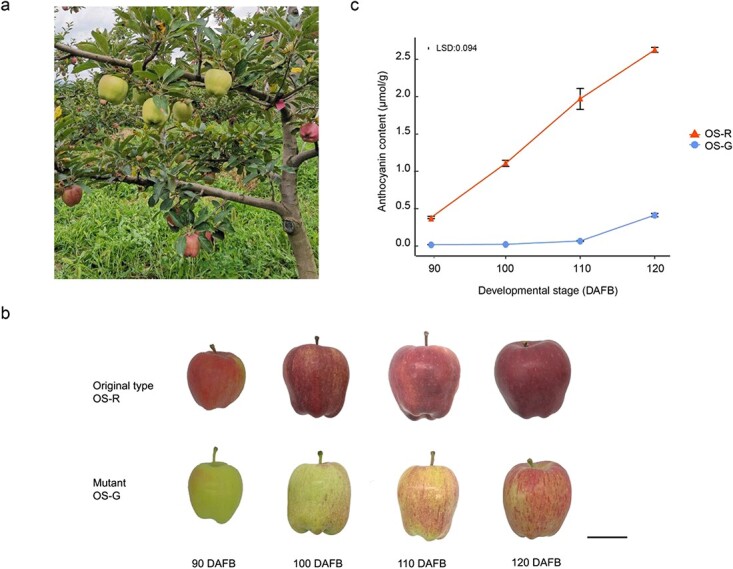
Fruit skin color and anthocyanin of original type and a bud sport of ‘Oregon Spur II’. **a** ‘Oregon Spur II’ tree showing a branch with reduced fruit color in 2020. **b** Fruits were collected at 90, 100, 110, and 120 DAFB from OS-R and mutant OS-G. Bar = 5 cm. **c** Comparison of anthocyanin content between OS-R and mutant OS-G at four developmental stages. The vertical bar represents the least significant difference at the 5% level of significance of three independent biological measurements, which was used for means comparison between the original type and mutant and time points (DAFB). Vertical bars at each developmental stage represent the standard error of the mean.

In an orchard, one branch of a mature ‘Oregon Spur II’ tree had green to pale red fruit at 90 days after full bloom (DAFB) in multiple years whereas other branches of the same tree had red fruit (Fig. 1a, Supplementary Data Fig. S1a). Moreover, the trees produced by grafting buds from the mutant branch also produced green fruit (Supplementary Data Fig. S1b). These data together suggested that the branch producing green fruit was a bud-sport mutant (named OS-G) whereas other branches showed the original type phenotype with red fruit (named OS-R). As fruit developed from 90 to 120 DAFB, the fruit skin of both OS-R and mutant OS-G showed progressive coloration (Fig. 1b). However, OS-G fruits had slightly turned red when OS-R fruits had become totally red at 100 DAFB. In addition, OS-R fruits were visibly more intensely red than OS-G fruits at 100, 110, and 120 DAFB. OS-G fruits still had patches of green fruit skin at maturity, 120 DAFB (Fig. 1b).

To characterize the difference between OS-R and OS-G fruit skin color, we analyzed anthocyanin content, CCI, *a*, *h*°, *b*, and *L*. These parameters were markedly different between OS-R and OS-G fruit at each developmental stage (Fig. 1c, Supplementary Data Figs S1c and S2). During fruit development, the values of anthocyanin content, CCI, and *a* gradually increased. These values were much higher in OS-R than in OS-G. On the contrary, the values of *h*°, *b*, and *L* gradually decreased in both OS-R and OS-G. These values were much lower in OS-R than in OS-G (Supplementary Data Fig. S2). Moreover, the fruits of the original type and mutant branches showed no significant differences in soluble sugar, soluble acid, or starch content, or fruit firmness (Supplementary Data Fig. S3).

### *De novo* assembly of ‘Oregon Spur II’ genome

**Figure 2:**
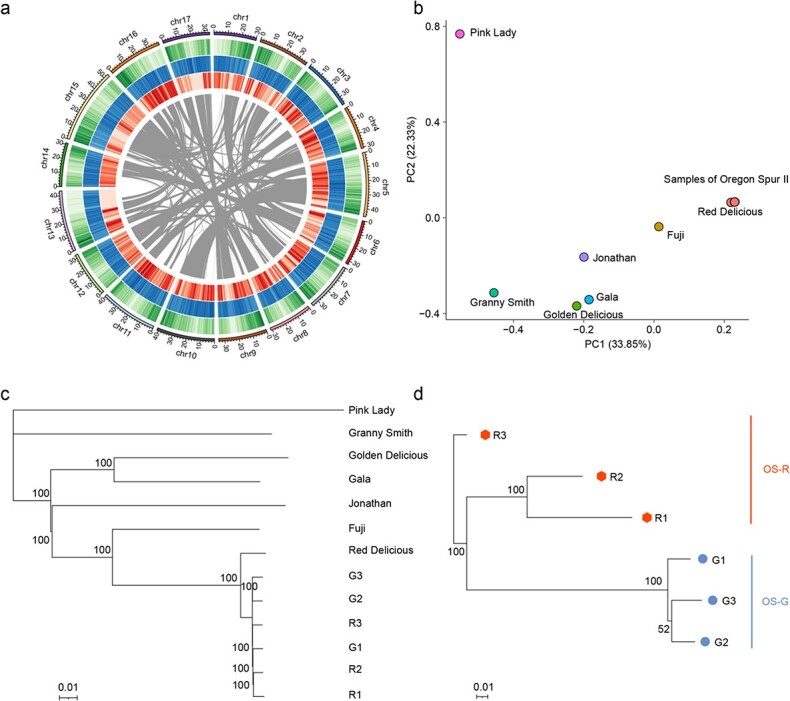
*De novo* assembly of the ‘Oregon Spur II’ genome and population structure analysis of ‘Oregon Spur II’ and seven *M. domestica* varieties. **a** Circular diagram depicting the characteristics of the ‘Oregon Spur II’ genome. The tracks from outer to inner circles indicate the following: chromosomes (chr), gene density (window size 500 kb); repeat density (window size 500 kb); SNP density (window size 500 kb); paralogous relationships between chromosomes. **b** PCA of ‘Oregon Spur II’ and seven *M. domestica* cultivars showed nearly identical genetic backgrounds between ‘Oregon Spur II’ and ‘Red Delicious’. **c** Phylogenetic tree of ‘Oregon Spur II’ and seven *M. domestica* cultivars based on genome-wide SNPs (neighbor-joining method with 1000-replicate bootstrap test). **d** Ontogenetic trees constructed using all 677 somatic SNPs identified among six samples of ‘Oregon Spur II’ (neighbor-joining method with 1000-replicate bootstrap test).

The genome of ‘Oregon Spur II’ was assembled using a combination of sequencing reads from Illumina HiSeq (186.57 Gb) and Oxford Nanopore Technologies GridION X5 (18.28 Gb with an average length of 15.64 kb) (Supplementary Data Table S1). The raw data of the Nanopore long reads was pre-corrected and assembled, which resulted in 809.1 Mb of sequences, with a contig N50 value of 1.59 Mb. After polishing and eliminating redundancies, the haploid contig assembly was 649.5 Mb with a contig N50 value of 2.04 Mb. Then, 624.99 Mb (96.36%) sequences of the haploid contig assembly were anchored onto 17 chromosomes. The final assembled ‘Oregon Spur II’ genome was 649.61 Mb (scaffold N50 value, 35.78 Mb; longest scaffold value, 53.62 Mb) (Fig. 2a, Table 1). Moreover, the ‘Oregon Spur II’ reference genome sequence showed strong collinearity and consistency with the ‘Gala’ genome sequence and the improved genetic map ‘iGLmap’ (Supplementary Data Figs S4 and S5).

**Table 1:** Statistics of the ‘Oregon Spur II’ genome assembly compared with previously published apple reference genomes.

	‘Oregon Spur II’	GDDH13 [7]	HFTH1 [6]	‘Gala’ [8]
Total assembly size (Mb)	649.61	643.2	660.5	652.4
Contig number	1724	2150	502	7560
Contig N50 (kb)	2041	620	6988	2317
Scaffold N50 (kb)	35 775	37 604	37 138	23 924
Percentage of sequence anchored on chromosome	96.10%	92.56%	98.78%	96.7%
Complete BUSCOs	97.10%	97.40%	98.20%	97.9%

Benchmarking Universal Single-Copy Orthologs (BUSCO) assessment showed that 97.1% of the complete genes could be detected in our assembly, which is similar to the values for published apple reference genomes (Table 1) [6–8].

### Genome annotation

Using a *de novo* approach, we identified ~380.7 Mb sequences being TEs that represented 58.57% of the ‘Oregon Spur II’ genome. Long terminal repeat (LTR) retrotransposons accounted for 42.22% of the genome as the most common type of TE. The most abundant LTR retrotransposons were the Gypsy elements (15.46%), followed by Copia elements (11.08%) (Supplementary Data Table S2).

In total, 45 982 high-confidence protein-coding genes were identified based on the evidence of *de novo* prediction, protein-based homology detection, and RNA sequence mapping (Fig. 2a, Supplementary Data Table S3). For functional annotation, >96% of the predicted genes were homologous with the known databases. Moreover, 17 480 (38.01%), 6312 (13.72%), and 30 937 (67.28%) annotated genes were assigned to biological process, cellular component, and molecular function according to Gene Ontology (GO) annotation (Supplementary Data Fig. S6).

### Population structure and kinship of major varieties

The sequence data from each Illumina and Nanopore library covered more than 43 and 9 times the apple genome, respectively (Supplementary Data Table S1). On the basis of genome-wide SNPs, principal component analysis (PCA) revealed that the samples of ‘Oregon Spur II’ were in close proximity to ‘Red Delicious’, and all six samples of ‘Oregon Spur II’ were almost the same (Fig. 2b). This result was confirmed by phylogenetic analysis that showed ‘Red Delicious’ and ‘Oregon Spur II’ samples clustered together (Fig. 2c). Moreover, kinship analysis showed that ‘Red Delicious’ and samples of ‘Oregon Spur II’ were clonally related (proportion of zero identical-by-state (IBS0) < .0007, kinship >.46). The kinship value between samples of ‘Oregon Spur II’ indicated a strong clonal relationship among these samples (IBS0 < .0003, kinship >.47) (Supplementary Data Table S4). The results of PCA, phylogenetic analysis and kinship analysis revealed that six samples of ‘Oregon Spur II’ were virtually identical and mostly close to ‘Red Delicious’. Moreover, the rate of IBS0 sites between ‘Red Delicious’ and OS-R was .0004–.0006, while the rate of IBS0 sites between OS-G and OS-R was .0001–.0002. These findings indicated that ‘Oregon Spur II’ was a somatic mutant of ‘Red Delicious’, and there were more genetic mutations between ‘Red Delicious’ and ‘Oregon Spur II’.

### Genetic variations in ‘Oregon Spur II’

A total of 677 somatic SNPs were identified between different sectors of the ‘Oregon Spur II’ tree (Supplementary Data Table S5). These somatic SNPs contained 475 heterozygous SNPs and 202 homozygous SNPs. The average numbers of somatic SNPs per sample were 226 and 344 for the original type and mutant branch, respectively. After normalization of genome size and sample numbers, the somatic mutation rate of the ‘Oregon Spur II’ tree was estimated to be 4.56 × 10^−8^ per base per year. Most of these SNPs (accounting for 55.98%) were distributed in the intergenic regions. Only 33 SNPs were located in the coding regions. Of these, 16 were non-synonymous SNPs that may affect protein properties (Supplementary Data Table S6). Furthermore, phylogenetic analysis using the 677 somatic SNPs classified the original type (OS-R) and mutant (OS-G) samples into two clades (Fig. 2d). Between these two clades, 253 somatic SNPs were identified. After 16 SNPs were re-analyzed by using Sanger sequencing, 14 SNPs were confirmed, indicating a validation rate of 87.5%. Five SNPs between OS-R and OS-G were non-synonymous and identified in five genes (*OS_007139*, *OS_008856*, *OS_018866*, *OS_028744*, *OS_037028*; Supplementary Data Table S7). Of them, *OS_007139* encodes an ERF transcription factor (Supplementary Data Table S7, Supplementary Data Fig. S7a). These non-synonymous SNPs may affect protein properties and thus fruit skin color.

Between different sectors of the ‘Oregon Spur II’ tree, 1212 somatic InDels were identified. Most of the InDels were located in intergenic regions, and 25 InDels were predicted to cause large effects, including codon changes and frame shifts. Small InDels (length <5 bp) accounted for 88.44% of the total InDels. Between OS-R and OS-G, 118 somatic InDels were identified. Four InDels were predicted to cause frame shifts in four genes: *OS_005743*, *OS_008922*, *OS_024777*, and *OS_043076* (Supplementary Data Table S7). Of them, *OS_024777* encodes an NB-ARC transcription factor (Supplementary Data Table S7). Moreover, one somatic structural variation (SV) (deletion) located in intergenic regions was identified between OS-R and OS-G (Supplementary Data Fig. S8a and b). The deletion was 7.3 kb upstream of a SCARECROW-LIKE gene (*OS_046436*) and 24 kb upstream of a MATE-type anthocyanin transporter gene (*OS_046435*) (Supplementary Data Table S7). Altogether, 11 genes were found to show key somatic mutations in OS-G.

### Characterization of the OS-R and OS-G transcriptomes and differentially expressed genes

**Figure 3:**
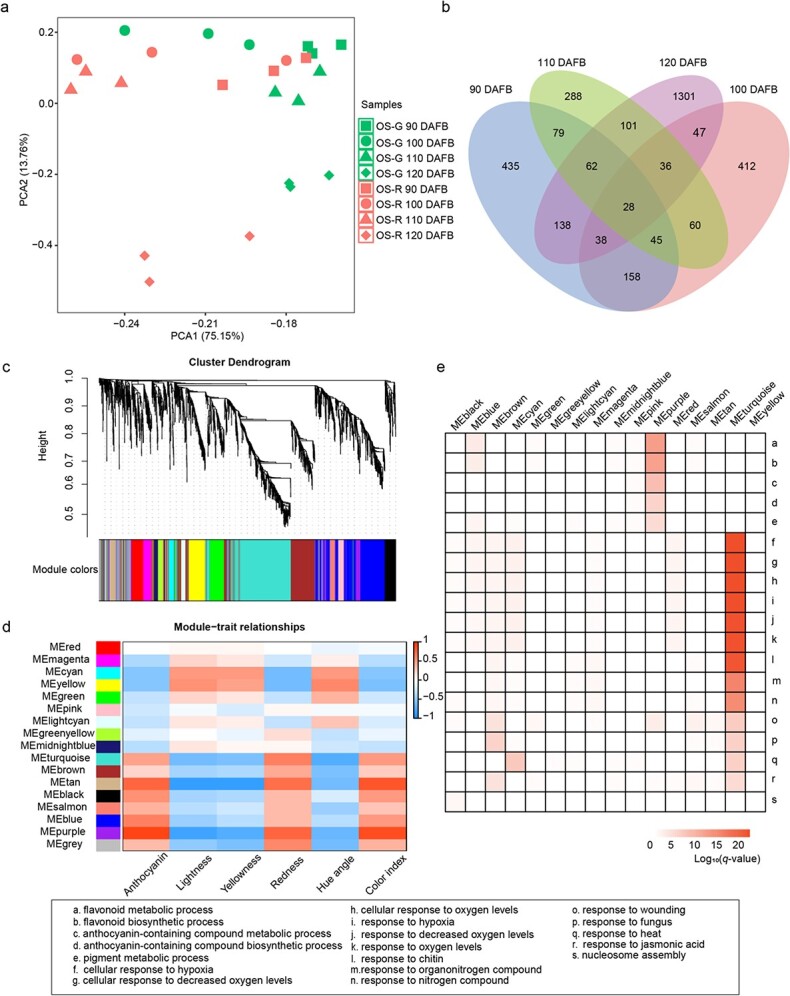
Transcriptome analyses showing DEGs between OS-R and mutant OS-G at four developmental stages. **a** PCA analysis of transcriptome data at four developmental stages of OS-R and mutant OS-G. **b** Number of DEGs between OS-R and OS-G at four developmental stages. **c** Hierarchical cluster tree showing co-expression modules identified by WGCNA. Each leaf in the tree is one gene. The major tree branches constitute 16 modules labeled by different colors. **d** Heat map showing weight correlations between 16 modules (left panel) and six phenotypic traits (bottom panel). The color scale at right shows module–trait correlation from −1 (blue) to 1 (red). **e** GO functional categories enriched by genes of different modules. Only significant categories (*q*-value <1e^−5^) are displayed.

A total of 628.62 million Illumina reads were generated from 24 libraries of OS-R and OS-G at four developmental stages with three biological replicates. Then, RNA-seq reads were cleaned and the resulting clean reads with Q20 and Q30 were mapped to the ‘Oregon Spur II’ reference genome. About 95.49% of clean reads were mapped to the reference genome (Supplementary Data Table S8). Generally, 28 620 genes expressed with transcripts per kilobase of exon per million mapped reads (TPM) >1.0 between OS-R and OS-G were used for further analysis. The expression values of three biological replicates were used for PCA analysis. The biological replicates of each group were clustered closely, and four categories could be distinguished according to developmental stages (Fig. 3a). In each category, OS-G and OS-R were separated. Moreover, OS-G and OS-R at 120 DAFB were far from samples at other developmental stages.

**Figure 4:**
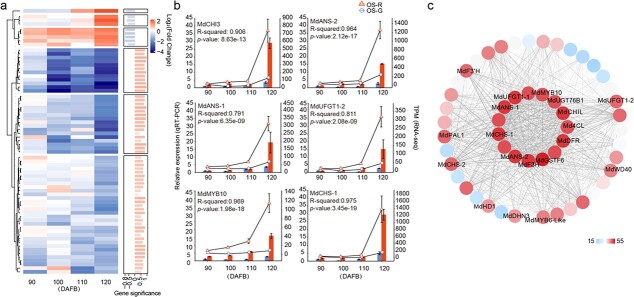
Analyses of DEGs in the ‘MEpurple’ module. **a** Heat map showing the log_2_ fold change of DEGs (OS-G/OS-R) from module ‘MEpurple’ at four developmental stages (S1, 90 DAFB; S2, 100 DAFB; S3, 110 DAFB; S4, 120 DAFB). The right bar plot shows the WGCNA gene significance for anthocyanin content (i.e. correlation with the trait). **b** Expression levels of six DEGs at four developmental stages were determined by qRT–PCR (columns, left *y*-axis) and RNA-seq (lines, TPM values on right *y*-axis). The *R*^2^ and *P* values of correlation analyses between qRT–PCR and RNA-seq expressions are indicated. **c** Cytoscape figure showing co-expressed genes with edge weight ≥0.1 in the ‘MEpurple’ module. The edge number of the genes ranges from 15 to 55 (color-coded by the scale on right from blue to red).

At each developmental stage, the transcriptome comparison between OS-R and OS-G was used to identify differentially expressed genes (DEGs). This allowed 4257 DEGs to be identified (3228 non-redundant), which included 680 genes differentially expressed at multiple stages (Fig. 3b). There were 1135 DEGs in the early stages, with 435 at 90 DAFB, 412 at 100 DAFB, and 288 at 110 DAFB, less than the DEGs observed at the last stage (1301 at 120 DAFB).

### Weighted gene co-expression network analysis

Weighted gene co-expression network analysis (WGCNA) was adopted to identify gene modules and key genes that may lead to the color change of fruit skin. A total of 3228 DEGs were used in the WGCNA analysis and clustered into 16 modules (Fig. 3c). Analysis of the module–trait relationships revealed that the ‘MEpurple’ module containing 69 genes was highly correlatedwith both total anthocyanin content (*r* = .92, *P* = 8 × 10^−10^), colorindex (*r* = .87, *P* = 3 × 10^−8^), and lightness (*r* = −.8, *P* = 2 × 10^−8^) and moderately correlated with redness, yellowness, and hue angle(Fig. 3d). The module ‘MEtan’ contained a total of 56 genes, whichwere highly associated with lightness (*r* = −.8, *P* = 3 × 10^−6^) and yellowness (*r* = −.8, *P* = 2 × 10^−6^) (Fig. 3d). Moreover, the other 14 modules did not seem to be associated with anthocyanin content or the other parameters associated with fruit skin color.

The results of GO analysis of cluster genes in the ‘MEpurple’ module showed that the GO terms were ‘flavonoid metabolic process’, ‘flavonoid biosynthetic process’, ‘anthocyanin-containing compound metabolic process’, ‘pigment metabolic process’, ‘regulation of flavonoid biosynthetic process’, ‘response to UV’, and ‘phenylpropanoid metabolic process’ (Fig. 3e and Supplementary Data Table S9). As flavonoid and anthocyanin have been previously reported to determine apple fruit color [31], genes in the ‘MEpurple’ module may play an important role in apple fruit skin for anthocyanin content to ensure coloration. However, the cluster genes in the ‘MEtan’ module were enriched in GO terms like ‘response to herbivore’, ‘terpenoid biosynthetic process’, and ‘terpene biosynthetic process’ (Fig. 3e and Supplementary Data Table S9). Thus, it could be seen that the genes in the ‘MEpurple’ module played a vital role in the coloration of apple fruit skin and were worthy of further analysis.

### Identification of genes involved in biosynthesis of anthocyanin

The ‘MEpurple’ module contained 69 genes, including 8 encoding transcription factors, such as MYB, TCP, HD-ZIP, and MADS transcription factors. We found several genes differentially expressed in the last three and in all developmental stages and the expression levels of these genes in OS-R were always higher than in OS-G (Fig. 4a). Meanwhile, the expression pattern of these genes coincided with the variation of anthocyanin content (Fig. 4a). The intra-modular hub genes with most connections in the ‘MEpurple’ module of the network were identified, including *MdMYB10* (*OS_027409*), *MdCHI3* (*OS_046031*), *MdMYB6-like* (*OS_039911*), *MdUFGT1-1* (*OS_046729*), *MdUFGT1-2* (*OS_022154*), *MdANS-1* (*OS_022201*), *MdANS-2* (*OS_042975*), *MdCHS-1* (*OS_040070*), *MdF3H* (*OS_012046*), *Md4Cl* (*OS_046747*), *MdDFR* (*OS_000222*), and *MdGSTF6* (*OS_030227*) (Supplementary Data Table S10). Their expression levels in OS-R increased during fruit development and were highest at 120 DAFB (Fig. 4b). Moreover, the expression of *MdMYB10* showed huge differences between OS-R and OS-G at four developmental stages. Notably, *MdMYB10* had a large number of connecting lines (edges) (Fig. 4c). The above results further showed the reliability of the WGCNA result and that *MdMYB10* as a hub gene in the module ‘MEpurple’ plays a key role in anthocyanin biosynthesis in apple skin.

### Analysis of genetic variations combined with gene expression analysis

To identify the causative genetic variations, the expression patterns of the 11 genes containing key somatic mutations were analyzed using the transcriptome data. Eight of them showed expression in apple skin (Supplementary Data Table S7). The expression pattern of the six genes containing a non-synonymous SNP did not show a clear difference between OS-R and OS-G (Supplementary Data Fig. S7b). These genes were *OS_007139* (ERF transcription factor), *OS_024777* (NB-ARC transcription factor), *OS_028744* (nucleoporin and receptor-like protein), *OS_008922* (TIR-NB-LRR), *OS_005743* (hypothetical protein), and *OS_037028* (*O*-fucosyltransferase) (Supplementary Data Fig. S7b). Of the two genes flanking the large SV, *OS_046436* (SCARECROW-LIKE) did not show a clear difference in expression pattern between OS-R and OS-G (Supplementary Data Fig. S8c). *OS_046435* (MATE-type anthocyanin transporter) showed a higher expression level in OS-R than in OS-G at the last two stages (110, 120 DAFB) of fruit skin development (Supplementary Data Fig. S8c). *OS_046435* was also co-expressed with *MdMYB10* in the ‘MEpurple’ module (Supplementary Data Table S10).

### Genome-wide examination of cytosine methylation in OS-R and OS-G

Epigenetic variations have been reported to cause stable changes in gene expression to generate sport mutants, such as DNA methylation [2, 13]. To investigate the differences in methylation levels between OS-R and OS-G, we sequenced DNA extracted from fruit skin sampled at 120 DAFB and generated >69 million (bisulfite sequencing) BS-seq reads for each of the four libraries (Supplementary Data Table S11). The clean reads were mapped to the ‘Oregon Spur II’ reference genome to analyze cytosine methylation (Supplementary Data Table S12). The correlation of the BS-Seq data for the OS-R and OS-G fruit skin in CG, CHG, and CHH contexts was presented in Supplementary Data Fig. S9, which shows that the biological replicates of each sample are clustered..

**Figure 5:**
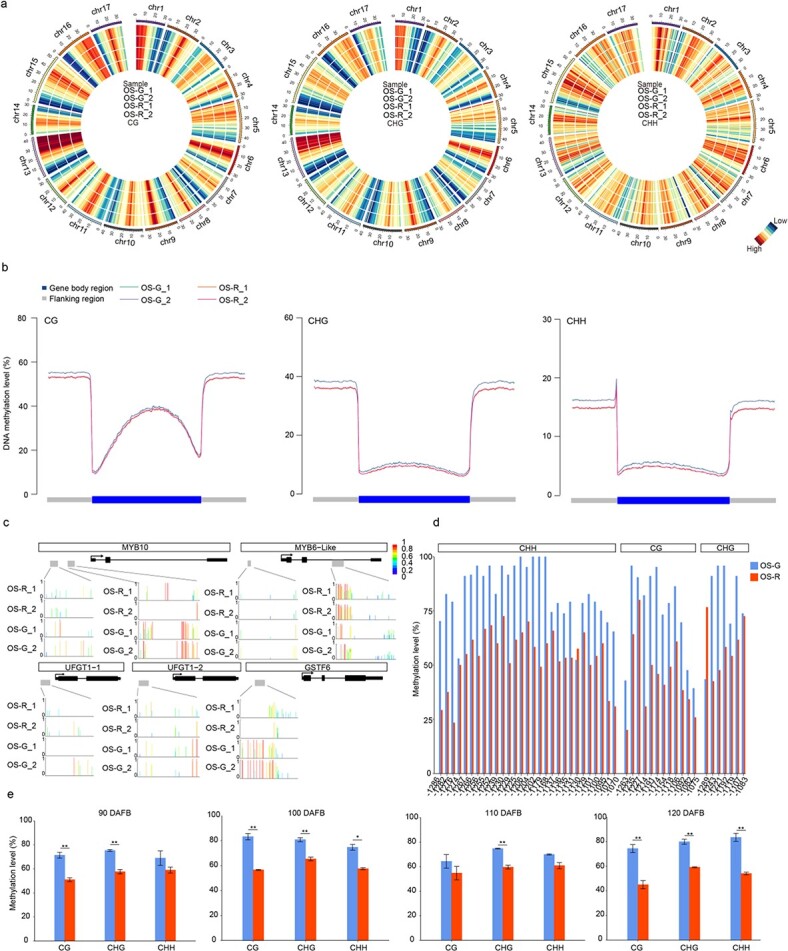
DNA hypermethylation was detected in mutant OS-G compared with OS-R. **a** Circos plots showing genome-wide DNA methylation levels at CG, CHG, and CHH sites. The four samples, OS-G_1, OS-G_2, OS-R_1, and OS-R_2, are arranged from outer to inner circle of the plots. Average methylation rates were calculated within 1-Mb windows. **b** Methylation levels (%) at CG, CHG, and CHH sites in gene bodies, 2 kb upstream of transcription start sites and 2 kb downstream of transcription termination sites were compared between OS-R and OS-G. **c** DMRs were identified in five anthocyanin-associated genes (*MYB10*, *MYB6-like*, *UFGT1-1*, *UFGT1-2*, and *GSTF6*) between OS-R and OS-G. The data in **a**, **b**, and **c** were generated by using whole-genome bisulfite sequencing of fruit skin of OS-R and OS-G at 120 DAFB. **d** Methylation levels of each cytosine in the *MdMYB10* promoter (−1254 to −1034) in OS-R and OS-G at 120 DAFB were determined using BSP. **e** Mean methylation levels of three biological replicates were calculated after the *MdMYB10* promoter region (−1254 to −1034) at 90, 100, 110, and 120 DAFB was analyzed using BSP. Asterisks (^*^P < .05, ^**^P < .01) indicate significant differences between OS-G and OS-R as determined by Student’s *t*-test.

To show the global DNA methylation levels, the average 5mC rate of 1-Mb windows through the genome was plotted as a heat map in CG, CHG, and CHH contexts respectively (Fig. 5a). The methylation levels of the genome were 60.91–62.49%, 43.6–45.44%, and 15.27–16.54% in the CG, CHG, and CHH contexts, respectively. Small differences were detected between OS-R and OS-G in CG, CHG, and CHH contexts. Then we compared the average methylation levels within gene bodies among the samples to examine DNA methylation patterns. The profiles of DNA methylation levels in the CG, CHG, and CHH contexts were similar to the typical pattern previously reported in apple [32], with the highest methylation levels in the flank regions (2 kb upstream of transcription start sites and 2 kb downstream of transcription termination sites), followed by the gene body region (Fig. 5b).

### Differentially methylated regions between the OS-R and OS-G

An Upset plot was used to present the distribution of differentially methylated regions (DMRs) in genes and promoter regions in CG, CHG, and CHH contexts. The OS-R_vs_G comparison group comprised 5283, 2276, and 2025 DMR-associated genes (DMR_genes) as well as 4554, 4452, and 5371 DMR_promoter_genes (genes of DMR-associated promoters) in the CG, CHH, and CHG contexts, respectively (Supplementary Data Fig. S10a). The methylation levels in each context of the DMRs were slightly lower in the fruit skin of OS-R than in that of OS-G (Supplementary Data Fig. S10b). The number of DMRs identified in different genic regions (promoter, 5′-UTR, 3′-UTR, exon, and intron) was different. In each context, there were more hypermethylated DMRs than hypomethylated DMRs in all regions (Supplementary Data Fig. S10c). Moreover, the 5′-UTR and 3′-UTR regions contained the fewest DMRs.

To further understand the function of the DMR_genes and DMR_promoter_genes, GO analysis was performed. The DMR_promoter_genes were enriched in various processes, including response to salt stress and phenylpropanoid biosynthetic process for the CG context (Supplementary Data Fig. S11a), flavonoid metabolic process and flavonoid biosynthetic process for CHG context (Supplementary Data Fig. S11b), and pigment biosynthetic process for CHH context (Supplementary Data Fig. S11c). The GO enrichment information regarding DMR_genes is provided in Supplementary Data Fig. S11d–f.

### Analysis of differentially methylated regions related to the anthocyanin pathway

We identified five DMR_promoter_genes and one DMR_gene related to anthocyanin content, including *MdMYB10* (CHG_hypermethylated_promoter, CHH_hypermethylated_promoter), *MdUFGT1-1* (CG_hypermethylated_promoter), *MdUFGT1-2* (CHG_hypermethylated_promoter), *MdMYB6-Like* (CHG_hypermethylated_promoter), *MdGSTF6* (CHH_hypermethylated_promoter), and *MdMYB6-Like* (CHH_hypomethylated_genebody) (Fig. 5c). On the basis of the transcriptome data, these genes were downregulated in OS-G, which had significantly higher DNA methylation levels than OS-R at 120 DAFB. Moreover, a CHG- and a CHH-type DMR were identified in the promoter of *MdMYB10*, located between −1254 and − 1034 and between −713 and − 518 upstream from the start codon, respectively. These results indicated that the DNA methylation levels in OS-G may inhibit the transcription of some anthocyanin-related genes that contribute to the coloration of apple fruit skin.

Methylation levels of the *MdMYB10* promoter were validated using the bisulfite sequencing PCR (BSP) approach. The region from −1289 to −1012 was used to detect the target 221-bp DNA fragment (−1254 to −1034) containing 29 CHH, 12 CG, and 7 CHG cytosine methylation sites (Fig. 5d). The average methylation levels in all three contexts (CHH, CG, and CHG) were significantly higher in OS-G than in OS-R at 120 DAFB (Fig. 5e). Moreover, the methylation levels in all contexts were always higher in OS-G than in OS-R at 90, 100, and 110 DAFB (Fig. 5e). Whole-genome bisulfite sequencing (WGBS) and BSP analyses both showed that the methylation levels in the promoter of *MdMYB10* were increased in OS-G compared with OS-R.

## Discussion

A genomics approach was used in this study to identify the causative mutation underpinning the fruit skin color degeneration of the apple cultivar ‘Oregon Spur II’. Based on a newly assembled high-quality reference genome of ‘Oregon Spur II’, genome-wide comparisons between the original type and a bud sport branch were made using sequence data of multiple genomes, DNA methylomes, and transcriptomes. These comparisons showed that hypermethylation of the *MYB10* promoter was most likely the cause of the reduced red coloration of fruit skins of the bud sport.

The ‘Oregon Spur II’ reference genome assembled in this study is a valuable addition to the previously assembled apple reference genomes. These previous apple reference genomes are derived from ‘Golden Delicious’ [7] and its descent cultivars ‘Gala’ [8] and ‘Royal Gala’ [33], or from ‘Hanfu’ [6]. The ‘Oregon Spur II’ reference genome is derived from a ‘Red Delicious’ bud sport. ‘Red Delicious’ has no pedigree relationships to ‘Golden Delicious’ (Fig. 2b and c) [34] and contributes a quarter of the genome of ‘Hanfu’ through ‘Fuji’ [6, 34]. Like ‘Golden Delicious’, ‘Red Delicious’ is another important cultivar that is extensively used as a parent in apple breeding [34]. The ‘Oregon Spur II’ reference genome is more suitable than the ‘Golden Delicious’ reference genomes for analyzing ‘Red Delicious’ descent populations. Multiple reference genomes of the same species are often used for genomics analyses. For example, reference genomes of ‘Golden Delicious’ and ‘Royal Gala’ have been used together to identify transposable elements responsible for *MYB* gene allele-specific expression and flower color variations [33]. Thus, the ‘Oregon Spur II’ reference genome may be used together with previous reference genomes to compare different alleles of the same gene.

The genome-wide identification of somatic mutations using the first tree showing the sport phenotype has several advantages over using a sport population. The latter has been widely used to identify somatic mutations in horticultural plants [11, 35]. Somatic mutations accumulate in horticultural plants over their life span [10]. Similarly, we observed the accumulation of somatic mutations in the apple tree over multiple years and the somatic mutation rate of 4.56 × 10^−8^ per base per year. This rate is much higher than that in other horticultural plants previously reported [10, 11]. Moreover, numerous genetic variants were observed between ‘Red Delicious’ and ‘Oregon Spur II’, indicating that somatic mutations have been accumulated since the separation of ‘Oregon Spur II’ from ‘Red Delicious’. The large number of somatic mutations that have been accumulated over the long time period of the development of a sport population have an adverse effect on the identification of the causative genetic variants for the sport phenotypes. In contrast, the sport branch has only been separated from the original-type branches for a much shorter time period and has accumulated a much smaller number of mutations. Therefore, the comparison between the sport and original-type branch of a young tree should be more effective than comparison between the original cultivar and its sport population for identifying the causative somatic mutations.

**Figure 6:**
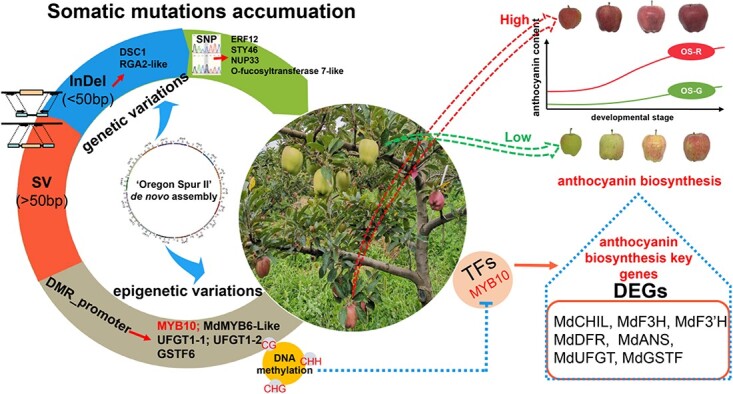
The epigenetic variations detected in ‘Oregon Spur II’ may contribute to the emergence of a green sport mutant. Five non-synonymous SNPs, four frame-shift InDels, and one SV were detected between OS-R and mutant OS-G. With current knowledge, we cannot postulate if genes containing one of these variants have a function associated with anthocyanin biosynthesis. However, hypermethylation regions were detected in the promoter of five genes known to be involved in anthocyanin biosynthesis. These hypermethylated promoters were also associated with reduced gene expression. We suggest that these epigenetic variations, in particular the one in the promoter of *MYB10* encoding the key transcription factor that activates anthocyanin biosynthesis, may underlie the reduced anthocyanin phenotype of the bud sport of ‘Oregon Spur II’.

Genetic and epigenetic variants can contribute to the bud sport phenotype that is transmitted by vegetative propagation [17, 36, 37]. This study first compared the genetic variants, including SNPs, InDels, and SVs, between the original type and the mutant branch (Fig. 6). Of the variants identified, only a few are located within the genes showing expression in fruit skin and can potentially affect gene expression or protein sequence (Supplementary Data Table S7). Two relevant genes are *OS_007139*, encoding an ERF transcription factor (Supplementary Data Table S7), and *OS_024777*, encoding an NB-ARC transcription factor (Supplementary Data Table S7). The homologs of the ERF and NB-ARC transcription factors are reported to affect the anthocyanin content [38, 39]. However, the somatic SNP is not located in conserved domains of *OS_007139* (Supplementary Data Fig. S7a), and *OS_024777* had a very low level of expression (Supplementary Data Fig. S7b). Moreover, one SV was found in the upstream region of the gene *OS_046435* (Supplementary Data Fig. S8a and b), encoding an anthocyanin transporter, MdMATE. The SV (a large deletion) seemed to reduce the expression of *OS_046435* in OS-G compared with OS-R at two of the four stages of fruit skin coloration (Supplementary Data Fig. S8b and c). As there was a color difference between OS-R and OS-G at all four stages tested, the reduction of *MdMATE* expression was only partially correlated with the reduction of anthocyanin accumulation. Given the unlikeliness that the above genetic variants are associated with reduced anthocyanin accumulation, we searched for epigenetic mutations that may play a role in reducing red coloration in the mutant fruits.

MdMYB10 is a key regulator of anthocyanin biosynthesis in apple. *MdMYB10* showed a co-expression pattern with anthocyanin pathway genes in ‘Oregon Spur II’, and the expression levels of *MdMYB10* and the pathway genes were repressed in OS-G compared with OS-R (Fig. 4b and c). These results are consistent with the reduced expression of *MdMYB10* and anthocyanin pathway genes in the yellow fruit skin sport of the red-skinned ‘Gala’ apple [13]. The methylation level of the *MYB10* promoter was higher in OS-G than in OS-R. This high level of methylation might cause the downregulation of *MYB10* expression, thus further reducing anthocyanin content. This is similar to previous results showing that a high methylation level of the *MYB10* promoter is associated with reduced *MYB10* expression and anthocyanin content in apple [13, 14] and pear [17]. The high level of methylation is associated with two DMRs. One is a CHH-type DMR between −713 and − 518 from the start codon (Fig. 5c), identified for the first time in this study. The other one is a CHG-type DMR between −1254 and − 1034 (Fig. 5c), which overlaps with a previously identified DMR, named MR3 (−1246 to −780), in the *MdMYB10* promoter [13]. MR3 is hypermethylated in the fruit skin of ‘Blondee’, a yellow sport of the red-skinned ‘Gala’ apple [13]. Similarly, the CHG-type DMR was hypermethylated in OS-G fruit skin at four fruit developmental stages (Fig. 5e). However, MR3 is associated with a much greater reduction of anthocyanin accumulation than the CHG-type DMR. This difference may be explained by the differences in methylation level and the precise location between MR3 and the CHG-type DMR, or the differences in genetic background between ‘Oregon Spur II’ and ‘Gala’. Altogether, these results indicate that the hypermethylated promoter of *MdMYB10* is most likely a causative epigenetic variant for the OS-G bud sport (Fig. 6).

In conclusion, this study assembled a high-quality apple reference genome and used it together with multi-omics tools for the identification of the causative genetic variants of the reduced anthocyanin content phenotype. After analyzing a number of genetic and epigenetic mutations, DMRs in the promoter of *MdMYB10* were considered to be responsible for the apple skin color reduction.

## Materials and methods

### Plant materials and DNA sequencing

This study used a tree produced in 2010 by grafting *M. domestica* cv. ‘Oregon Spur II’ buds to an *M. baccata* rootstock. The tree was grown in a commercial orchard in Tianshui, Gansu province, China. A branch of this tree was found to produce fruit with strongly reduced red coloration of fruit skin in 2016. This branch was named OS-G and referred to as mutant, while the original type branches were named OS-R. The buds of the OS-G branch were grafted to other apple trees in 2016. The fruit skin color of the initial OS-G branch and those produced in 2016 were phenotyped in 2018, 2019, and 2020. Leaves from terminal (1), middle (2), and basal nodes (3) of shoots on the original-type branch and the mutant branch were collected in 2020 for DNA isolation using a previously described method [40]. With these DNA samples, six Illumina sequencing libraries with an insert size of 350 bp were separately constructed. These libraries were sequenced using the Illumina HiSeq 2500 platform. Moreover, two Nanopore libraries with an insert size of 20 kb were separately constructed with DNA from the leaves collected from the terminal nodes of OS-G and OS-R using a published method [41]. These libraries were sequenced using the GridION X5 platform. Fruit skin tissues were collected in 2020 at 90, 100, 110, and 120 DAFB from the original-type and mutant branch with three replicates (two fruits per replicate), and were used for extraction of anthocyanin, DNA, and RNA. The extracted DNA and RNA were used for methylome and transcriptome analyses, respectively.

### Color parameters

Chromatic analyses of fruit skin color were executed following the Commission International de l’Eclairage (CIE) system. The parameters, including lightness (*L)*, redness (*a*), and yellowness (*b*), were measured using a chroma meter (Konica Minolta CR400, Osaka, Japan). The other parameters, hue angle (*h°*) and color composition index (CCI), were determined according to published methods [42, 43]. Each fruit was measured at four evenly distributed equatorial sites.

### Measurements of anthocyanin content

The apple fruit skin was ground with liquid nitrogen and extracted for anthocyanin using a previously described method [44]. The absorbance of the solution was measured using a microplate reader at 530, 620, and 650 nm. The final anthocyanin concentration was calculated based on the following equation: optical density (OD) = (A530 − A620) − [0.1 × (A650 − A620)] [45].

### Measurements of soluble sugar, soluble acid, fruit firmness, and starch content

The apple fruit flesh was extracted for soluble sugar, soluble acid, fruit firmness, and starch content using a previously described method [46].

### Genome assembly

The ‘Oregon Spur II’ genome was assembled using whole-genome sequencing reads generated from six Illumina and two Nanopore libraries (Supplementary Data Table S1). The raw Illumina sequencing reads were cleaned using Trimmomatic (version 0.39) to remove adaptors and unpaired-end reads, with the parameters: LEADING:3 TRAILING:3 SLIDINGWINDOW:4:15 MINLEN:36. NECAT was used for raw Nanopore read correction and genome assembly with specified parameters GENOME_SIZE = 630 M, MIN_READ_LENGTH = 2000 and other default parameters [47]. After that, the assembled contigs were polished using NextPolish together with paired Illumina reads and corrected Nanopore reads [48]. Then, the polished contigs were filtered by purge_dups to remove junk sequences and redundant haplotigs [49]. Furthermore, purged contigs were corrected, ordered, and oriented using Ragtag [50] based on the published high-quality genome of ‘Gala’ [8]. Finally, we used ALLMAPS [51] to estimate collinearity between the assembled genome sequences and a previously improved genetic map, iGLmap [52].

### Genome annotation

Extensive *de novo* TE Annotator (EDTA) was used to annotate the repetitive sequences, construct the TE library, and filter raw TE candidates. Then the TEs were searched and identified by mapping the sequence to the EDTA library with RepeatMasker [53].

We used both *de novo* gene prediction and homologous gene prediction along with RNA-seq-assisted prediction to annotate the genome assembly based on the MAKER annotation pipeline [54]. The *de novo* gene prediction was performed by using BRAKER2 with transcriptional data downloaded from NCBI [55, 56] and generated in this study, which incorporates Augustus [57, 58]. Additionally, the transcriptional data of different tissues from ‘Red Delicious’ and ‘Oregon Spur II’ were assembled using Hisat2 and StringTie [59, 60]. After that, MAKER2 was used to generate a consensus gene set by combining the result of *de novo* gene prediction, assembled transcripts, and protein sequences (GDDH13, https://iris.angers.inra.fr/gddh13/the-apple-genome-downloads.html; SwissProt, https://www.uniprot.org/taxonomy/33090). The Annotation Edit Distance (AED) score was used to filter the gene set. BUSCO was used for the evaluation of annotation completeness with eudicotyledons_odb10 [61].

We performed functional annotation by mapping the ‘Oregon Spur II’ protein sequences to SwissProt and NR databases using diamond (e-value ≤1e−5). The InterProScan (version 5.36) was used to annotate the motifs and domains with the parameter -appl ProDom,PRINTS,Pfam,smart,PANTHER [62]. Based on the corresponding InterPro entry, the GO annotations were assigned.

### Mapping DNA sequence reads and identifying genomic mutations

The clean paired-end reads of six samples from ‘Oregon Spur II’ and seven published *M. domestica* cultivars [63] were mapped to the ‘Oregon Spur II’ reference genome via BWA-MEM with -M and default parameters [64]. Then the BAM files were sorted by SAMtools, and PCR duplicates were marked by Picard. The HaplotypeCaller of the Genome Analysis Toolkit (GATK, version 4.1.0) was used to retrieve all genotype information from each sample [65]. The pipeline of somatic mutation calling previously described [10] was used to predict the candidate somatic SNPs and InDels of ‘Oregon Spur II. For generating candidate somatic SNPs, we filtered SNP sites to remove those with low variant quality, low read depth, high missing rate, within 150 bp from InDels’ positions, and identical mutation allele in the original type. PCR and Sanger sequencing were employed to validate somatic SNPs. The somatic SNPs and InDels were annotated using SnpEff [66] based on the gene model of the ‘Oregon Spur II’ reference genome. SNPs were assigned to intergenic or different genic regions based on their locations.

SVs were called based on the ‘Oregon Spur II’ reference genome and the Illumina whole-genome resequencing data of the six ‘Oregon Spur II’ samples using Manta v1.6.0 [67], LUMPY v0.2.13 [68], and DELLY2 v0.7.7 [69]. The SVs called by different methods were merged by SURVIVOR v1.0.3 [70] with the following parameters: 1000 3 1 1 0 0. Comparison of SV genotypes was employed to predict the somatic SVs between OS-R and OS-G. GridION sequencing data were analyzed using NGMLR v0.2.7 and sniffles v1.0.12 [71] to confirm the candidate somatic SVs. We further manually investigated all candidate SVs by using the Integrative Genomics Viewer (IGV) [72] to review the mapping states across all samples.

### Kinship and phylogeny analysis

To investigate the relationship between different samples of the ‘Oregon Spur II’ tree and other *M. domestica* varieties, PCA and kinship analysis were carried out based on the population SNPs using plink (v1.9) [73] and KING (v2.2.3) [74]. The phylogenetic analyses were carried out using VCF2Dis and Phylip [75]. The population SNPs and somatic SNPs were used to construct the phylogenetic tree, respectively.

### RNA isolation and sequencing

Total RNA was extracted according to a published method [40] and used for RNA-seq and qRT–PCR analyses. RNA-seq libraries were prepared using the NEBNext Ultra RNA Library Prep Kit. From these libraries, paired-end sequence reads of 150 nucleotides were generated using the Illumina HiSeq 2500 platform.

### RNA-seq read mapping and differential expression analysis

The raw reads were cleaned as described above. After that, the clean reads were mapped to the ‘Oregon Spur II’ reference genome using Hisat2 (v2.2.1) with default parameters [59]. Then, read counts of each gene were performed with featureCounts (v2.0.1) [76]. Gene expression was then quantitatively estimated by TPM. DESeq2 (v1.30.0) was employed to select the genes with differential expression through the parameters |log2foldchange| ≥ 1 and *P* < .05 [77]. OS-R and OS-G were compared at each developmental stage.

### Weighted gene co-expression network analysis and functional enrichment analysis

DEGs were selected between OS-R and OS-G at each developmental stage for follow-up analysis. The highly co-expressed gene modules were inferred from the DEGs using WGCNA [78]. A total of 3228 genes were used for WGCNA network construction and module detection with default parameters (power β of 9) as described before [13]. Then, a Pearson correlation test was used to evaluate the correlation of eigenvalues of each module with anthocyanin content in the 24 samples. The genes of the most significant module with WGCNA edge weight ≥0.5 were visualized using Cytoscape_3.1 [79].

We used clusterProfiler (v3.18.1) for statistical analysis of GO enrichment, and a significance level (*q*-value <.05) was employed [80].

### Gene expression analysis by qRT–PCR

Using qRT–PCR analysis, we compared the relative expression levels of DEGs between OS-R and OS-G samples (with three biological replicates) at each developmental stage as described before. The details of the qRT–PCR primers are given in Supplementary Data Table S13.

### Bisulfite sequencing and data analysis

Four BS-seq libraries were constructed using genomic DNA extracted from fruit skin tissues collected from OS-R and OS-G at 120 DAFB, including two biological replicates for each fruit type. The DNA was treated as described before for constructing the library [32]. Finally, the BS-seq libraries were sequenced on the Illumina HiSeq 2500 platform.

The raw reads were cleaned by removing adapters and low-quality reads before being mapped to the ‘Oregon Spur II’ reference genome with Bismark (v0.23.0) and default parameters [81]. Global methylation rates in CG, CHG, and CHH contexts were analyzed using CGmaptools [82]. Differential methylation between OS-R and OS-G was analyzed using DMRfinder (v0.4) [83]. The DMRs were filtered with a threshold of delta mC > .15 and false discovery rate (FDR) < .05. ChIPseeker (v1.26.0) [84] was used to annotate the DMRs and clusterProfiler (v3.18.1) was used to identify significantly enriched GO terms among the DMR-associated genes (*P* < .05).

A BSP assay was conducted to analyze the methylation levels in the *MdMYB10* promoter in fruit skin of OS-R and OS-G at the four developmental stages as previously described [13]. The details of BSP primers are given in Supplementary Data Table S13.

### Statistical analysis

Statistical analyses were performed using SPSS 20.0 (SPSS, Chicago, IL, USA). Three replicates were used to analyze variance and least significant differences. The experimental data were analyzed using Student’s *t*-test and the correlation of two variables was evaluated using Pearson correlation analysis.

## Acknowledgements

We are grateful to Professor Xiaofei Wang for help in revision of the manuscript. This work was financially supported by the National Key Research and Development Project (2018YFD1000101, 2019YFD1001803), the Shaanxi Apple Industry Science and Technology Project (2020zdzx03-01-04), the Studying Abroad Personnel Science and Technology Activity Fund Project of Shaanxi Province (2020-07), the Cyrus Tang Foundation, the Tang Scholar by Cyrus Tang Foundation and Northwest A&F University, and the China Apple Research System (CARS-27).

## Author contributions

D.Z. and J.L.Y. designed the research. Y.L., N.A., C.P.Z., and X.K.Z. collected the samples. J.J.M., X.H.G., and M.Z.L. performed the experiments. Y.L., L.T., and L.B.X. analyzed the data. Y.L., D.Z., and J.L.Y. wrote the manuscript. M.M.T. revised the manuscript. All authors participated in the research and approved the final manuscript.

## Data availability

All the raw data, as well as whole-genome, whole-genome bisulfite, and transcriptome sequencing reads have been deposited in the Genome Sequence Archive (Genomics, Proteomics & Bioinformatics 2021) at the National Genomics Data Center (Nucleic Acids Res 2021), China National Center for Bioinformation/Beijing Institute of Genomics, Chinese Academy of Sciences (GSA: CRA005304) and are publicly accessible at https://ngdc.cncb.ac.cn/gsa. Genome assembly and annotation data have been deposited at GSA under accession number GWHBJED00000000 and are publicly accessible at https://ngdc.cncb.ac.cn/gwh.

## Conflict of interest

The authors declare that they have no conflict of interest.

## Supplementary data

Supplementary data is available at *Horticulture Research* online.

## Supplementary Material

supp_data_uhac179
