# Supplementary material for: Multi-omics analyses reveal MdMYB10 hypermethylation being responsible for a bud sport of apple fruit color
Source: Hortic Res. 2022 Aug 29;9:uhac179. doi: 10.1093/hr/uhac179 (PMC9627520; doi:10.1093/hr/uhac179)
Supplement: supp_data_uhac179 [file supp_data_uhac179.zip › supp_fig.docx]

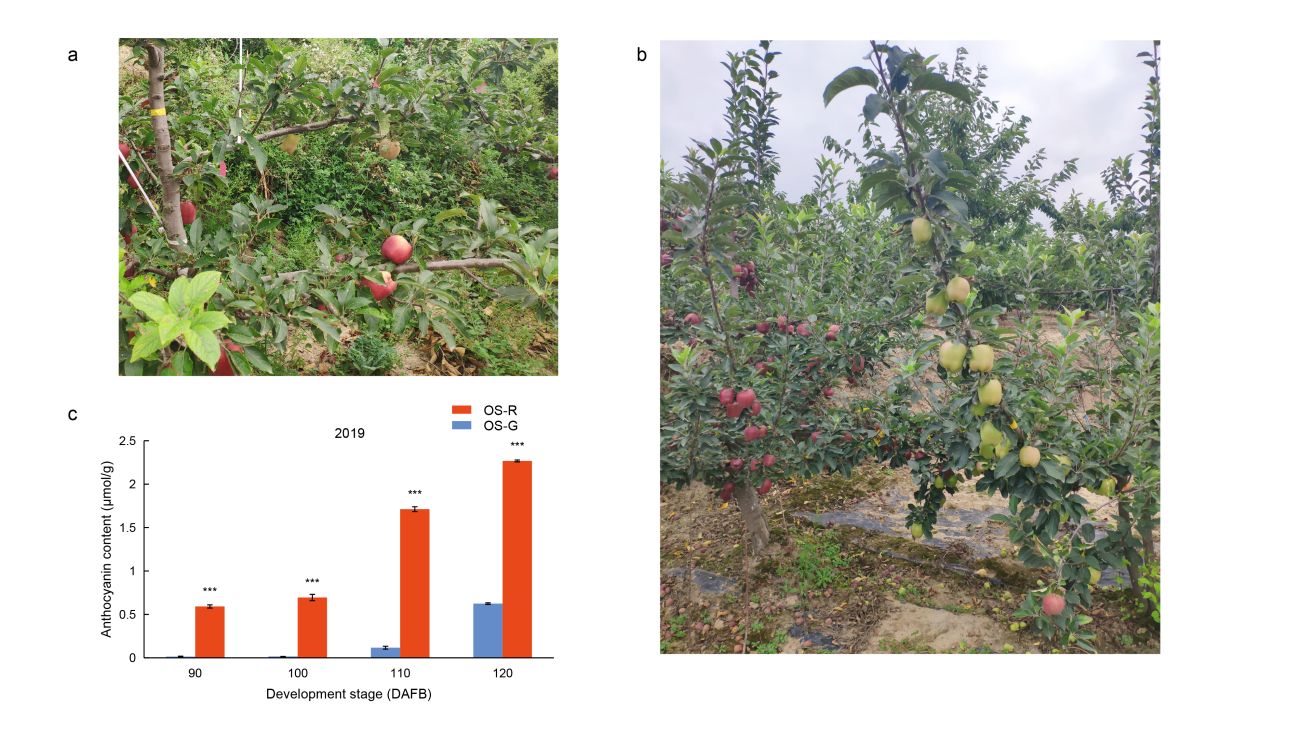


**Supplementary Figure S1. Fruit skin color and anthocyanin of wild-type (WT) and a bud sport of ‘Oregon Spur II’.** a. A photograph taken in 2019 shows an ‘Oregon Spur II’ tree with a mutant branch (circled) producing fruit with reduced red coloration. b. A photograph taken in 2021 shows a normal Oregon Spur II’ tree (left) and a mutant tree (right) produced by grafting a bud from the mutant branch of a to a rootstock in 2017. c. Comparison of anthocyanin content between the WT (OS-R) and mutant (OS-G) at four developmental stages. Error bars indicate standard deviation. Asterisks ‘***’ (P<0.001) indicate significant differences between OS-G and OS-R as determined by Student's t-test


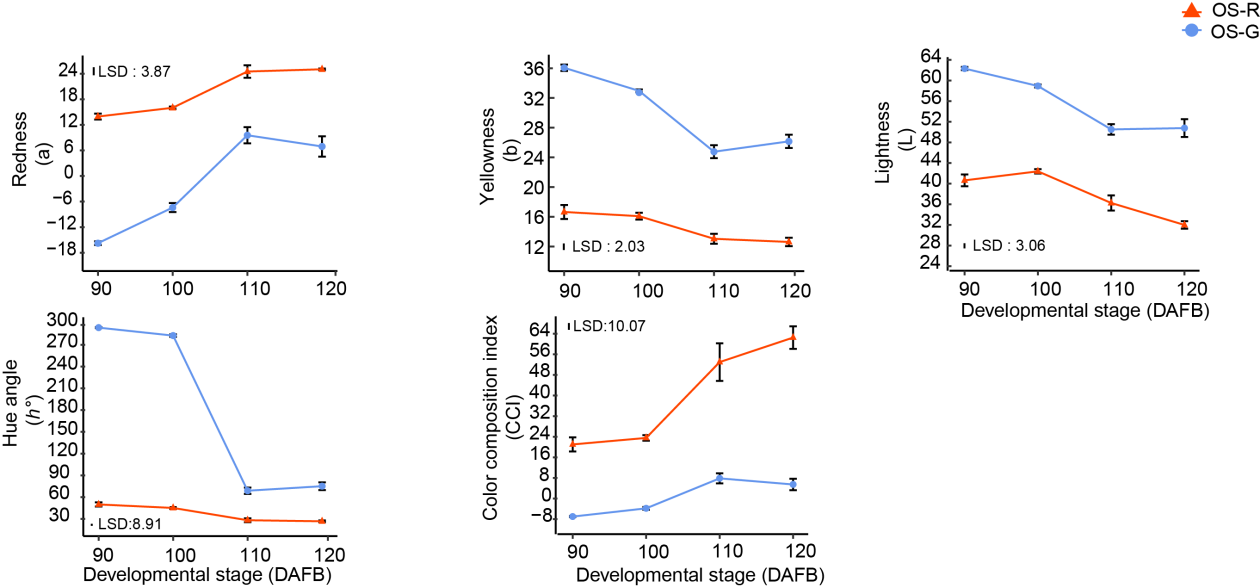


**Supplementary Figure S2. Comparison of redness, yellowness, lightness, hue angle and color composition index between the WT OS-R and mutant OS-G at four developmental stages.** The vertical bars represent the least significant difference (LSD) at 5% level of significance, of three independent biological measurements, which was used for means comparison between the WT and mutant and time points (DAFB). Vertical bars at each developmental stage represent the standard error of the means (SEM).


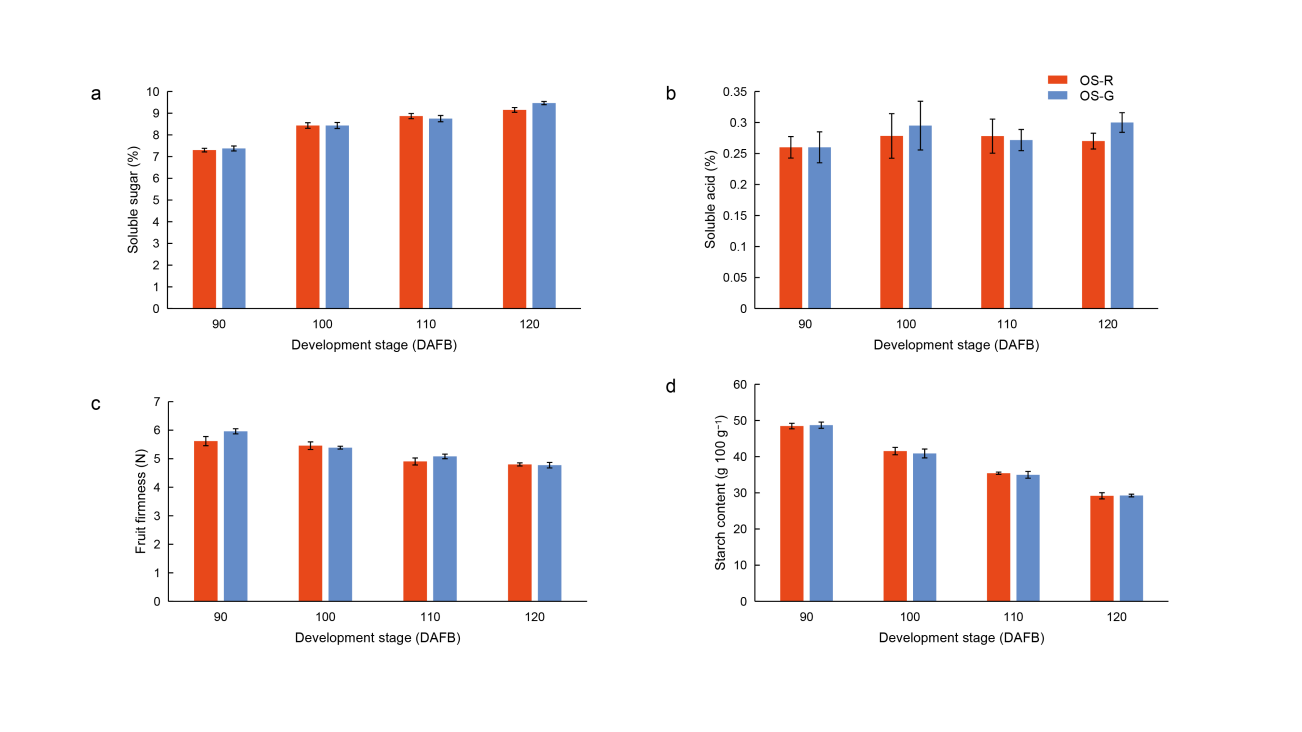


**Supplementary Figure S3. The soluble sugar, soluble acid, fruit firmness, and starch content in fruit flesh of the WT (OS-R) and mutant (OS-G) branch was analyzed at four developmental stages in 2019.** Error bars indicate standard deviation.


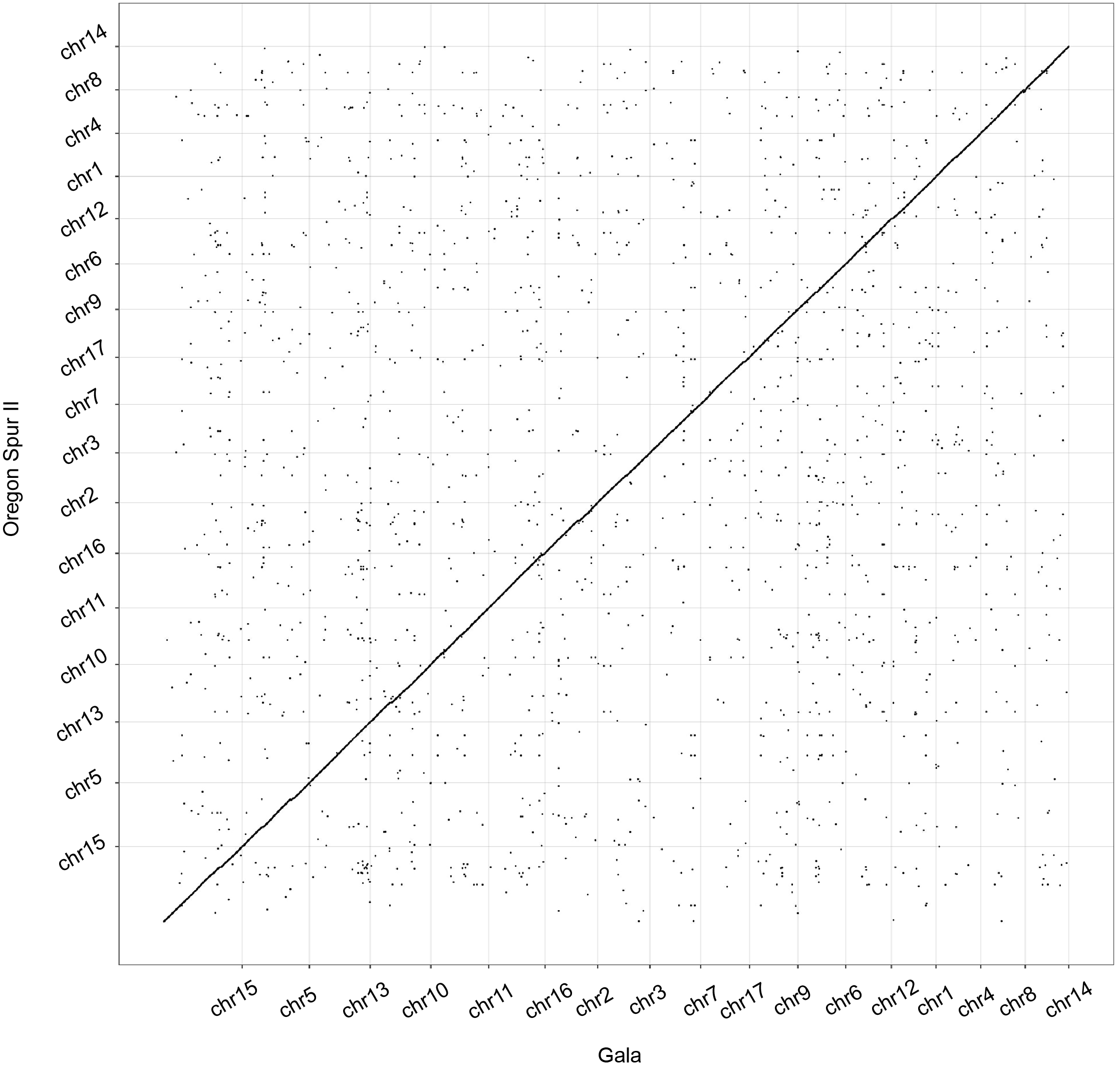


**Supplementary Figure S4. Genome collinearity between the ‘Oregon Spur II’ and ‘Gala’.** The x-axis corresponds to the ‘Gala’, and the y-axis corresponds to the ‘Oregon Spur II’.


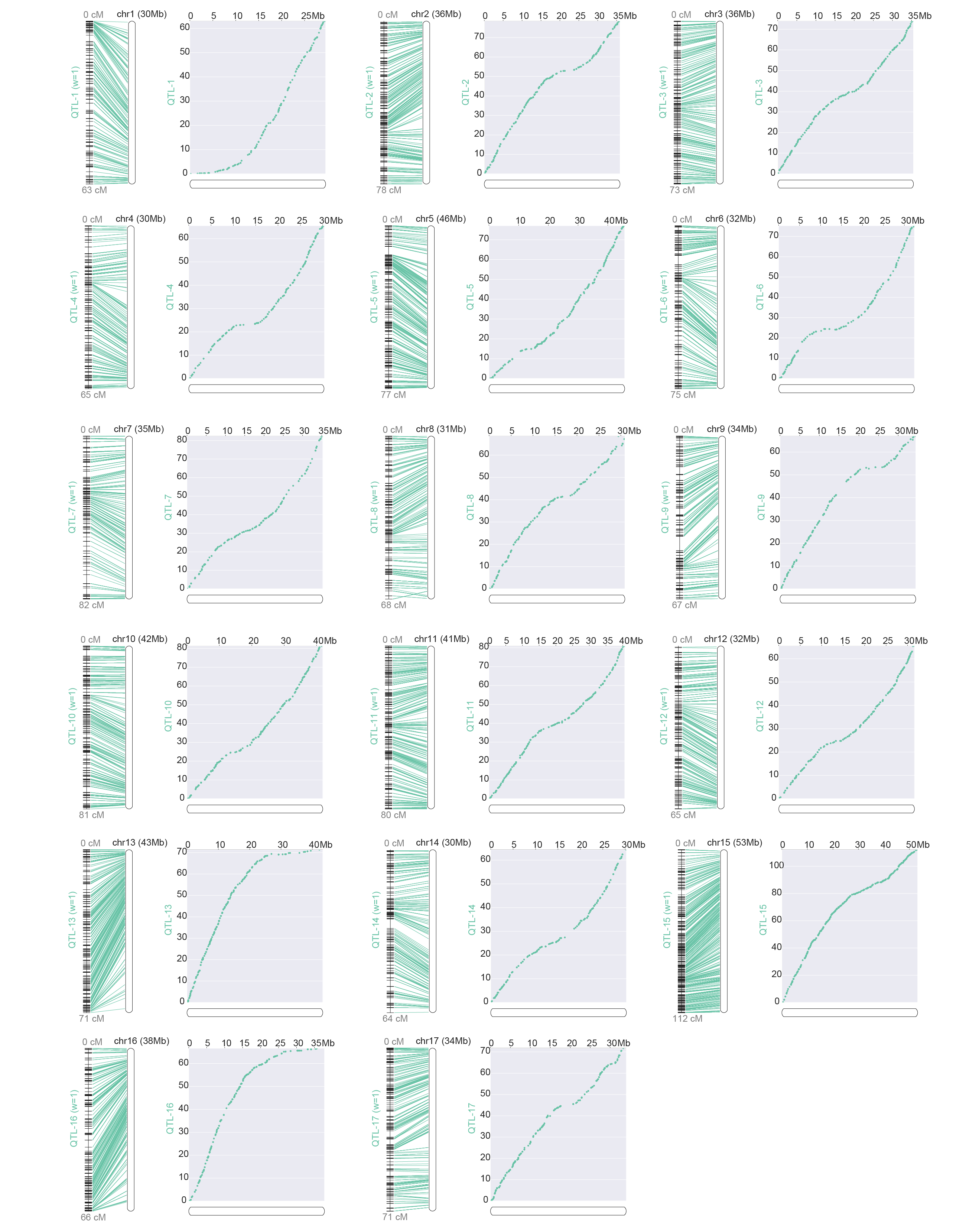


**Supplementary Figure S5. Collinearity between pseudo-chromosomes of ‘Oregon Spur II’ and ‘iGLmap’.** The high-density genetic map is shown on the left and the pseudo-chromosome (the middle panel) is shown on the right.


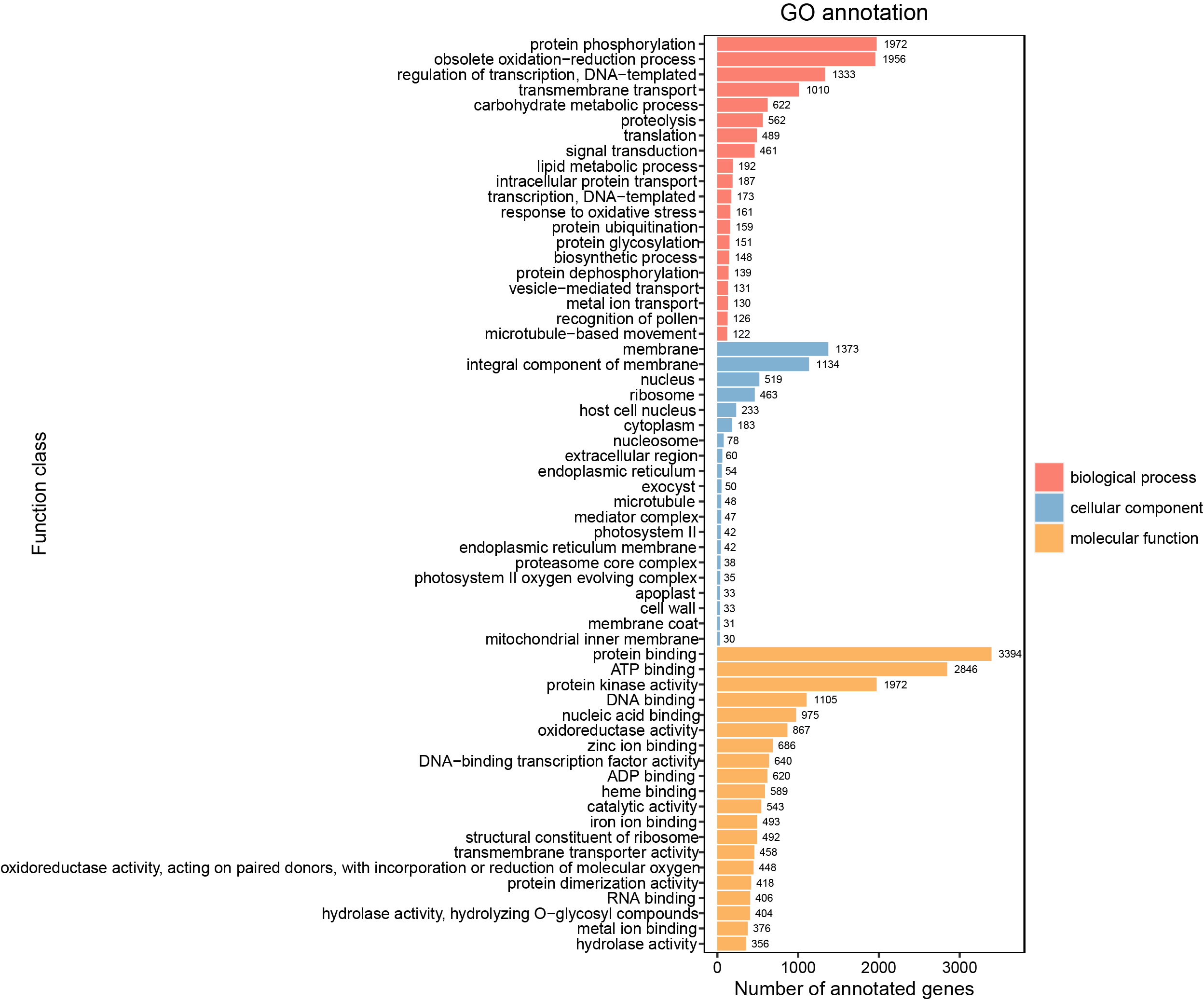


**Supplementary Figure S6. Gene ontology categories of the annotated genes.** The genes were annotated in three main categories: biological process, cellular component and molecular function (y-axis). The x-axis represents the count of genes identified in this study.


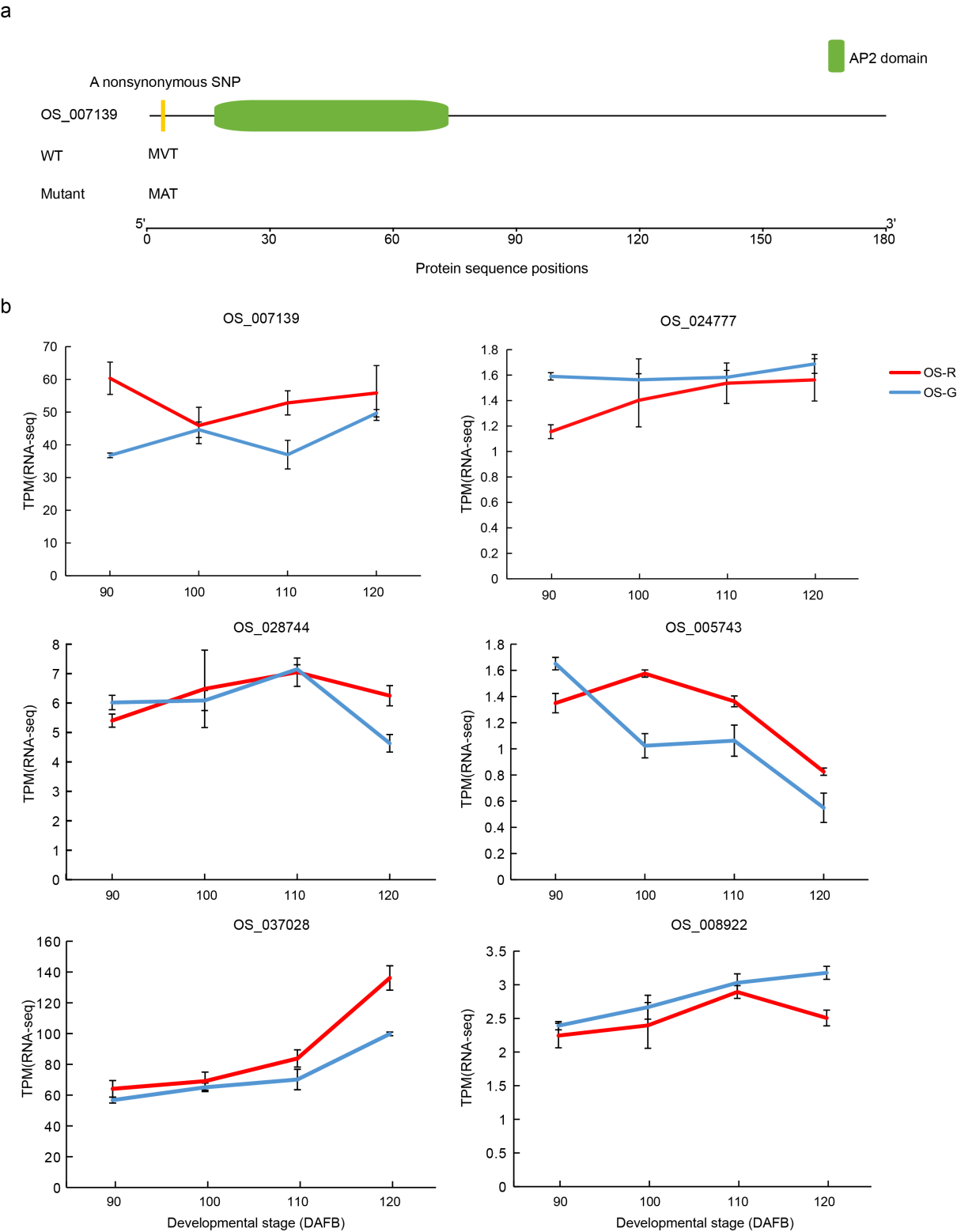


**Supplementary Figure S7. The domain architecture and expression of genes with candidate somatic mutations.** a. The second amino acid sequence of OS_007139 was altered by a nonsynonymous SNP. b. The expression levels of six genes with a nonsynonymous SNP at four developmental stages were determined by RNA-seq. Error bars indicate standard deviation.


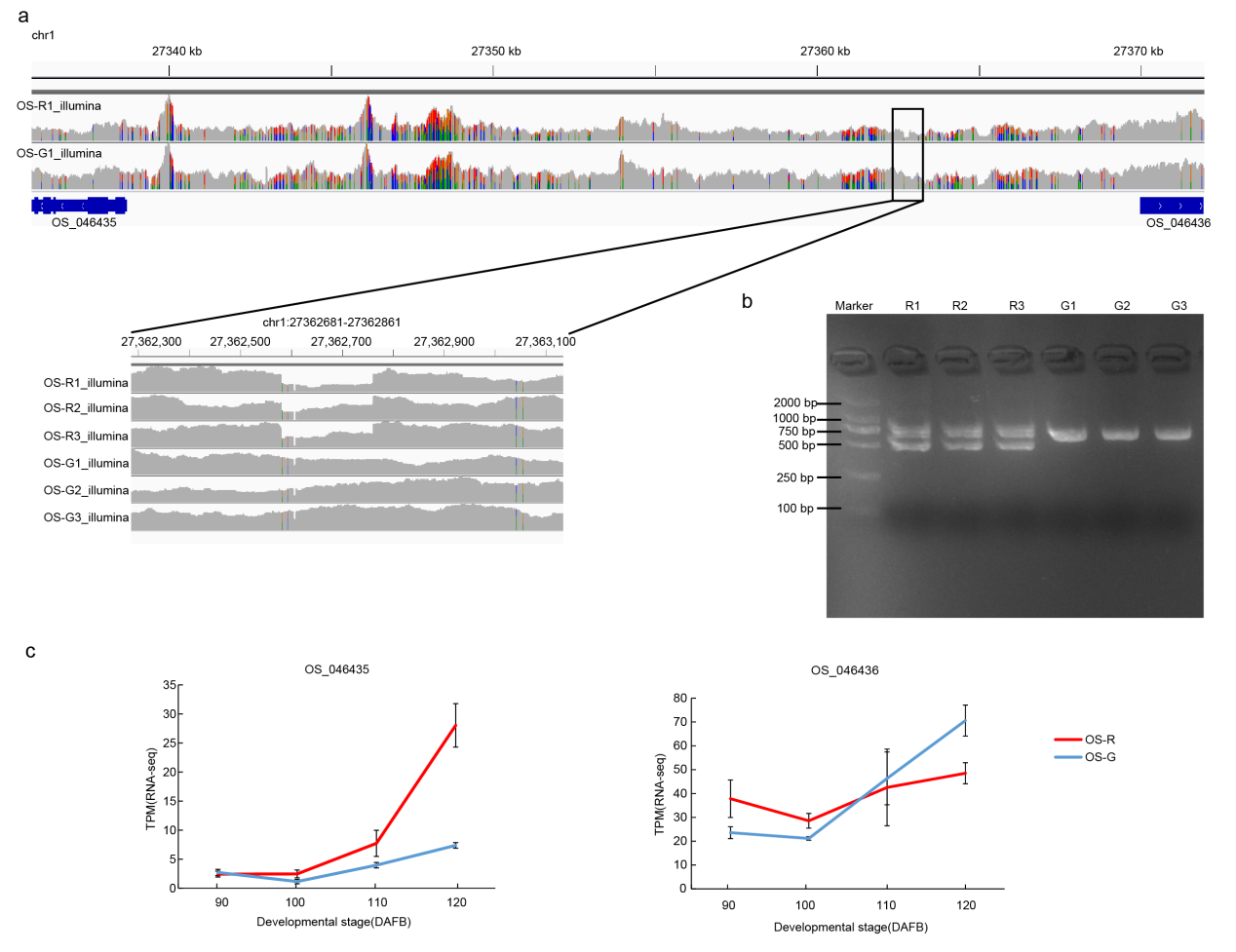


**Supplementary Figure S8. The validation of somatic SV between WT OS-R and mutant OS-G.** a. Validation the insertion in mutant OS-G by Illumina data in IGV. b. PCR confirmation of the somatic insertion in mutant OS-G. c. The expression levels of two genes located in the flanking region of the somatic insertion at four developmental stages were determined by RNA-seq. Error bars indicate standard deviation.


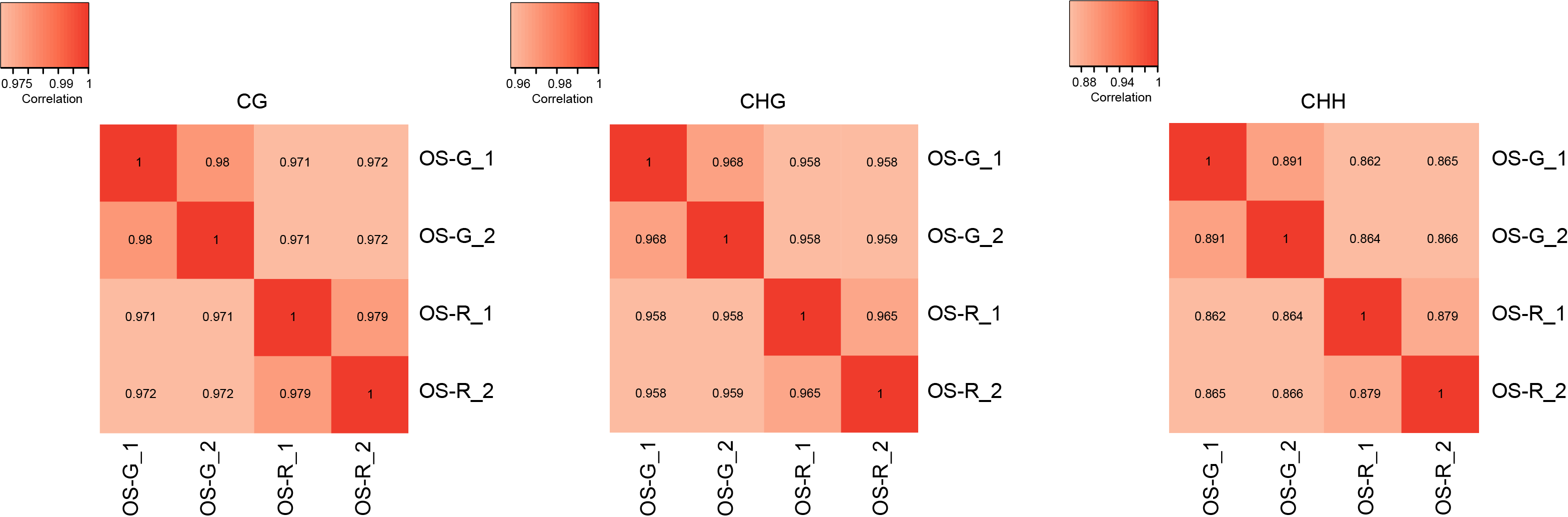


**Supplementary Figure S9. Correlation of the bisulfite sequencing data for the OS-R and OS-G.** Correlation of DNA methylation levels in the CG, CHG and CHH contexts.


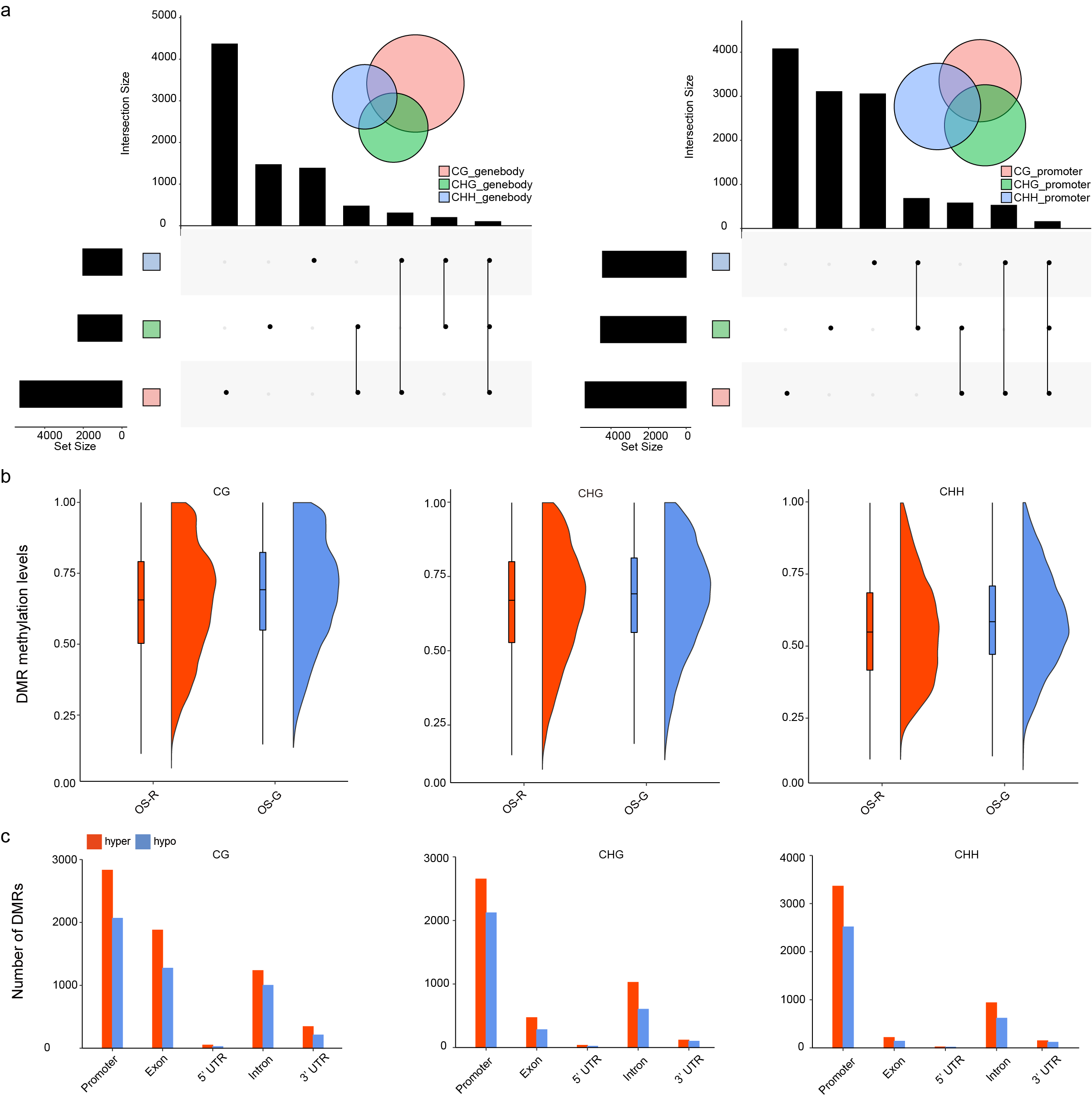


**Supplementary Figure S10. Differentially methylated regions (DMRs) and DNA methylation levels in WT OS-R and mutant OS-G.** a. Upset analysis of genes with DMRs in the CG, CHG, and CHH contexts in the gene-body regions (DMR_genes) as well as in the promoter regions (DMR_promoter_genes) between WT OS-R and mutant OS-G. b. Methylation levels in the CG, CHG, and CHH context in the DMRs of WT OS-R and mutant OS-G. c. Number of DMRs associated with the hyper/hypomethylated (OS-G/OS-R) sequences in the promoter, exon, 5’UTR, intron, 3’UTR.


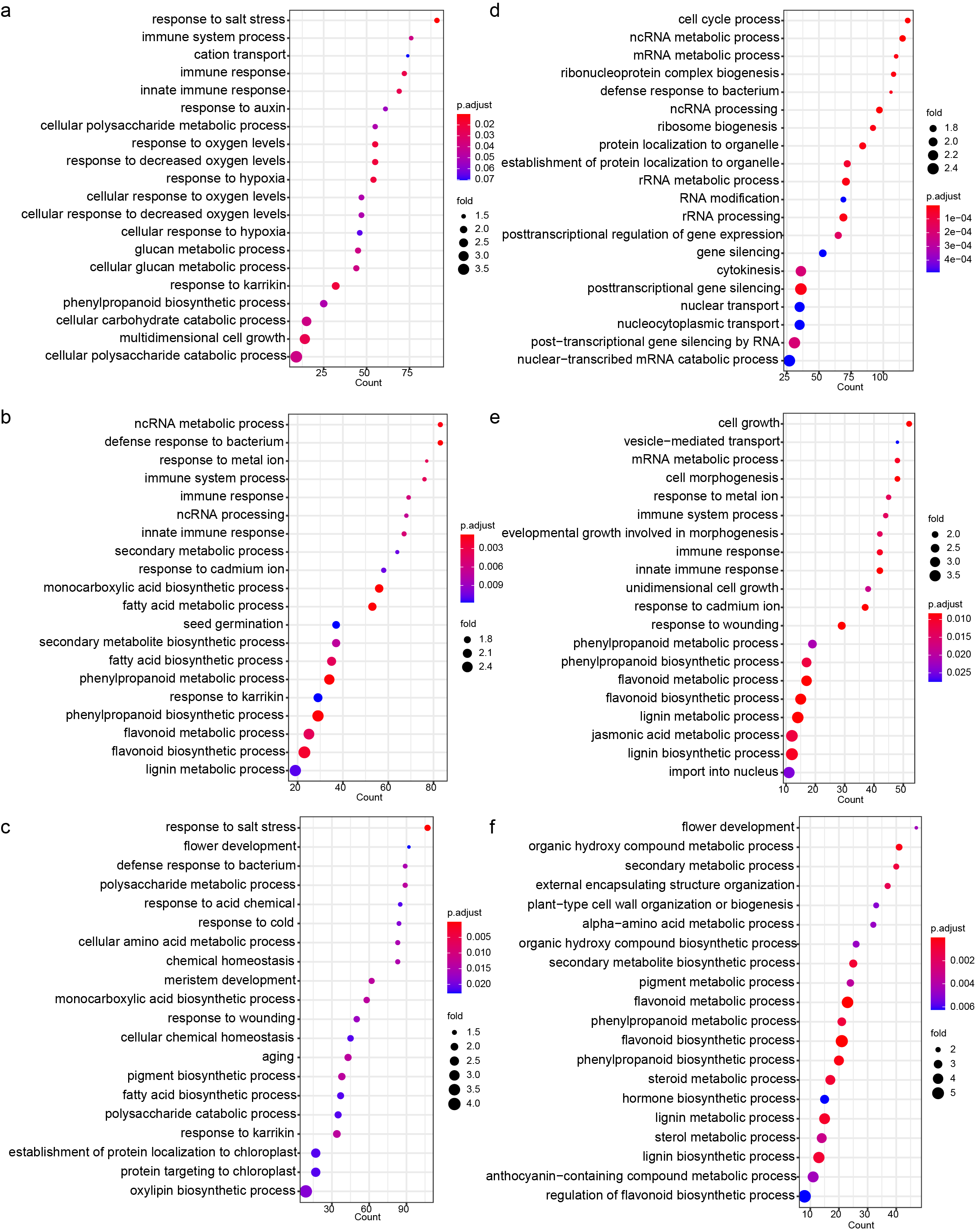
**Supplementary Figure S11. Gene ontology enrichment analysis of the DMR associated genes in the CG, CHG, and CHH context.** a, b, c. Details regarding the pathway enrichment for the DMR_promoter_genes in the CG, CHG and CHH context. d, e , f. Details regarding the pathway enrichment for the DMR_genes in the CG, CHG, and CHH context.
